# Lived experiences of individuals with cystic fibrosis on CFTR-modulators

**DOI:** 10.1186/s12890-022-01825-2

**Published:** 2022-01-21

**Authors:** Annelise Page, Aaron Goldenberg, Anne L. Matthews

**Affiliations:** 1 TriHealth, 10506A Montgomery Rd., Cincinnati, OH 45242 USA; 2 Case Western Reserve University, 10900 Euclid Ave., Cleveland, OH 44106 USA

**Keywords:** Cystic fibrosis, CFTR-modulators, Lived experiences, Quality of life, Career, Psychosocial, Family planning, Identity, Relationships, Ivacaftor, Lumacaftor, Lexacaftor, Tezacaftor

## Abstract

**Background:**

CFTR-modulators are a category of drugs that facilitate trafficking and opening of the abnormal CFTR protein in individuals with cystic fibrosis (CF) who have certain genetic mutations. Clinical trial data show that individuals taking CFTR-modulators have increased or stable lung function (FEV_1_) as well as reduced frequency of pulmonary exacerbations. There are no data on whether CFTR-modulators influence psychosocial aspects of the lives of individuals with CF. The purpose of this qualitative study was to explore how the introduction of CFTR-modulators has affected individuals’ lived experiences outside of clinical health variables; that is, to explore whether there is a relationship between using CFTR-modulator drugs and the psychological and social aspects of the lives of individuals with CF, including: career, relationships, family planning and psychological functioning.

**Methods:**

Eight men and women with CF ages 24–32, with a history of taking any approved CFTR-modulator for at least six months, were recruited from an adult CF center. A semi-structured interview guide was used to interview the participants. The data were coded using a grounded theory approach with an iterative methodology.

**Results:**

Four themes emerged from the data: stability, identity, potentiality, and hope.

**Conclusions:**

Although these themes cannot be generalized to all individuals with CF, this study provides preliminary data for how CFTR-modulators may influence an individual with CF’s outlook on life and that these individuals are feeling hopeful about the future.

## Background

Cystic fibrosis (CF) is the most common life-limiting autosomal recessive genetic disease in the United States with an incidence of 1 in 2500. The disease-causing gene involved in CF is the cystic fibrosis transmembrane conductance regulator (*CFTR*), which encodes a gating channel responsible for the transport of chloride ions (Cl^−^) across the cell membrane. Mutations in *CFTR* cause an abnormal transport of chloride ions across the cell membrane leading to dehydration of the organs. In the lungs this leads to a loss of airway surface liquid making mucus thicker and inhibiting the cilia from clearing the airways properly [1].

Historically, therapies for individuals with cystic fibrosis targeted the symptoms of the disease rather than the underlying disease-causing mechanism; however, recent treatments for cystic fibrosis have shifted from targeting the symptoms of CF to targeting its underlying defect. Pharmacological agents known as CFTR-modulators are intended to target the various defects in the CFTR protein that lead to disease. Individuals with cystic fibrosis who have specific mutations are eligible for these therapies. Current FDA approved CFTR-modulators include: ivacaftor (Kalydeco^®^), lumacaftor/ivacaftor (Orkambi^®^), tezacaftor/ivacaftor (Symdeko^®^), and elexacaftor/tezacaftor/ivacaftor (Trikafta^®^). It is important to note that this study was conducted in 2018, before the FDA approval of elexacaftor/tezacaftor/ivacaftor. Therefore, none of the participants in this study were prescribed elexacaftor/tezacaftor/ivacaftor at the time of their interview.

The majority (85–90%) of the CF population is eligible for one of these CFTR-modulators. The drugs available at the time this study was conducted (ivacaftor, lumacaftor/ivacaftor, and tezacaftor/ivacaftor) have been shown to lower the frequencies of pulmonary exacerbations and improve lung function (FEV_1_) at six months post treatment initiation [2–4]. Improvement in physical health of individuals with cystic fibrosis is important because it increases life expectancy and allows for an individual to lead a healthier life. Since modulators have only been on the market for a short period of time, individual lived experiences of those who are taking these drugs have not been explored outside of measurements of physical health.

A study conducted by Elsas et al. [5] explored prenatal genetic counselors’ attitudes about CFTR-modulators and the extent to which counselors were comfortable incorporating the effects of CFTR-modulators on individuals with CF into a counseling session. One hundred and fifty-seven prenatal genetic counselors were surveyed about how they thought a CFTR-modulator might influence an individual with CF’s physical health, psychological and emotional health, social functioning and personal goal fulfillment, treatment burden, and life expectancy. The participants surveyed in this study thought that psychological and emotional health as well as social functioning and personal goal fulfillment were the least likely to be impacted by CFTR-modulators. One of the counselors surveyed commented: “the reason I would not discuss a lot of the psychological and social health aspects is because there is no research yet that shows the drug improves psychological health and social functioning” [5, p. 69]. This revealed a gap in the literature regarding whether these therapies improve psychological health and social functioning.

One qualitative study in the literature aimed to address this gap by assessing the psychological and social impact of CFTR-modulators as well as the hopes and fears for the future of young adults with CF. Adults with CF ages 18–29 were interviewed using a semi-structured interview guide with questions about the participants’ aspirations and fears. Five themes emerged from the interviews: perceptions of living with unpredictable health and fear of death and dying; hopes for normality; hopes for a normal relationship and/or marriage; hopes for having children; and hopes for a normal work life. The four areas of an individual with CF’s life that were explored in our study: career, relationships, family planning, and psychological functioning were derived from this study conducted by Higham et al. [6].

There are published data exploring how individuals with CF experience their careers, relationships, family planning, and psychological functioning. Some of the literature suggests that psychological health is a better predictor for an individual with CF having a successful career rather than lung function [7, 8], while other studies show that constant absences from work due to illness related to CF results in less career satisfaction for these individuals [9]. The literature shows that there are different variables that affect romantic relationships for individuals with CF including constant hospitalizations and partners not being able to cope with premature death associated with cystic fibrosis [6]. Despite the stress that an individual with CF and a partner may experience, an individual with CF who has a partner is more likely to have a better quality of life [10].

Men and women with CF face different obstacles to starting a biological family. Greater than 95% of men with CF are infertile due to azoospermia caused by an absent vas deferens [11]. Men with CF may turn to artificial reproductive technologies such as in vitro fertilization (IVF) to have families. Mortality risk factors for pregnant women with CF are like women with CF who are not pregnant [12]. CFTR-modulators do not correct male infertility since the vas deferens is congenitally absent, but they may affect the lung function of a woman with CF considering pregnancy.‬ Men and women with CF also have similar concerns about family planning that are more psychological rather than physical. Data show that men and women with CF are hesitant to start families because of the possibility of leaving their child without a parent. Some individuals with CF decide to have children earlier in their life than they originally planned to spend more time with their children [6].

Psychological functioning is an important predictor of an individual’s quality of life. Poor psychological health in individuals with CF is associated with a lower quality of life [13]. Individuals with CF who are optimistic about the course of their disease have been found to have less health-related stress in their lives [14]. Some studies show that individuals with cystic fibrosis are not at more risk to have mental illness such as anxiety and depression than their peers [15], while other studies have shown that symptoms of depression are prevalent among young adults with CF [16].

While there are data demonstrating how CFTR-modulators affect lung function, weight gain, and frequency of pulmonary exacerbations there are not sufficient data on how these drugs may positively or negatively affect other areas of an individual’s lived experience. The aim of this study was to explore whether there is a relationship between CFTR-modulator drugs and the psychological and social aspects of the lives of individuals with cystic fibrosis including: career, relationships, family planning, and psychological functioning.

## Methods

### Study design

To understand the lived experiences of young adults with cystic fibrosis on CFTR-modulators this study used a qualitative methodology based on principles of grounded theory. The goals of qualitative research are: description, discovery, and understanding. Grounded theory is an approach that serves as a series of guidelines for how a researcher should collect and analyze qualitative data. This approach does not rely on a previous framework for interpreting the data; rather it relies on the data itself to construct theories [17].

### Participants and recruitment

This study took place in 2018. Participants were eligible for this study if they met the following criteria: (1) man or woman diagnosed with cystic fibrosis, (2) age 18–34, (3) taking any FDA approved CFTR-modulator (lumacaftor/ivacaftor, tezacaftor/ivacaftor, or ivacaftor) for at least six months, and (4) English-speaking individual. Participants were excluded from the study if they were a woman who was currently pregnant and was determined to have a high-risk pregnancy due to fetal abnormalities or maternal health complications unrelated to their CF, or if they had a significant life event or change unrelated to their CF that could affect how they view their: career, relationship, family planning, and/or psychological functioning (this was determined by the recruiter).

Participants were recruited from University Hospitals Cleveland Medical Center (UHCMC) Cystic Fibrosis Center by the Adult Program Director. The recruiter approached participants during their regularly scheduled visit to the CF clinic. If the participants expressed interest in participating in the study, they completed a recruiter form with their contact information, which CFTR-modulators they had taken and were currently taking, and the best time of day to be contacted. The recruiter also guided the participant through the consent form and had them provide written consent. The recruiter then sent the recruiter form and the informed consent document to the researcher who contacted the participants for a phone interview.

### Data collection

Interviews were conducted using a semi-structured interview guide (“Appendix 1”). The interview guide consisted of open-ended questions with follow up questions to allow participants to expand on their responses. The interview guide covered the following topics: health, career, relationships, family planning, and psychological functioning. Interviews were conducted over the phone and recorded with permission from the participant. Interviews ranged in length from 17 to 43 min, with an average time of 30 min. The site’s Institutional Review Board approved this study and all study materials.

### Data analysis

The interviews were recorded and transcribed verbatim by the researcher. The participants’ personal identifiers were removed from the transcripts. The first step to analyzing the data was coding the interviews. The coding process used a grounded theory approach with an iterative methodology. The researcher used constant comparative analysis to code the data. This is an inductive process where data from interviews are constantly reanalyzed and compared to newly collected data to generate themes. Data analysis began with line-by-line coding followed by focused coding of the first few interviews. For the additional interviews more broad themes and codes were explored with focused coding. Coding was done electronically using the software program Dedoose. From the codes identified patterns were determined and themes were formed based on clusters of codes. All the transcripts were evaluated for the themes. A separate researcher reviewed one transcript with the primary coder to review the codebook and confirm that there was consistency with how the codes were assigned to the text for that single interview.

## Results

### Demographics of the sample

Twelve participants who met the inclusion criteria were recruited from UHCMC CF Center. The recruiter conducted the informed consent process for all twelve potential participants and obtained written informed consent. The researcher attempted to contact each participant a maximum of three times and left voicemails with the permission of the participant. Four out of the twelve participants could not be contacted. These four individuals were females ranging in age from 20 to 33. The final sample consisted of four men and four women who were contacted and completed the interview (Table 1). Both “participant 3” and “participant 4” had previously been taking lumacaftor/ivacaftor and had transitioned to tezacaftor/ivacaftor three months prior to their interview. These two participants met inclusion criteria based on their use of two approved CFTR-modulators for at least six months total. Participants ranged in age from 24 to 33 years. The data are presented in four themes: (1) stability, (2) identity, (3) potentiality, and (4) hope.

**Table 1:** Demographic data of participants

Participant	Sex	Age range	CFTR-modulator at time of participation	Duration of modulator listed	Work status	Relationship status	Children (Y/N)
Participant 1	F	25–30	Tezacaftor/ivacaftor	6 mos	In school	In a relationship	N
Participant 2	M	25–30	Tezacaftor/ivacaftor	8 mos	Not Working	Married	Y
Participant 3	M	25–30	Tezacaftor/ivacaftor	3 mos	Full-time	Single	N
Participant 4	M	20–25	Tezacaftor/ivacaftor	3 mos	Not Working	Single	N
Participant 5	M	25–30	Tezacaftor/ivacaftor	7 mos	Full-time	Married	N
Participant 6	F	30–35	Tezacaftor/ivacaftor	9 mos	Full-time	Married	N
Participant 7	F	30–35	Tezacaftor/ivacaftor	8 mos	Not working	In a relationship	N
Participant 8	F	30–35	Lumacaftor/ivacaftor	3 yrs	Part-time	In a relationship	N

### Theme 1. Stability

The theme of “stability” refers to how participants perceived their physical health to be the same or improved since beginning the CFTR-modulator. Participants were asked about their current health status and if they noticed any significant changes to their health since they began taking the CFTR-modulator. Throughout the interviews, participants used the word “stable” when describing their physical health since they began to take the CFTR-modulator. For some participants, stability in their health was described by stable lung function (FEV_1_). One participant noted that:

“Um, just, you know, uh, well there’s always the side effects, which definitely were worse with Orkambi^®^, I remember that. Um, uh, but once those passed, um… less coughing, less mucus. Um, better… feels like you have more FEV_1_ and usually that’s borne out with the test results.”

(Participant 3, M)

Some participants also noted less frequent exacerbations since beginning the CFTR-modulator.

“I haven’t been… um, had any exacerbations, um, or needed any antibiotic treatment in the past… I’d say eight months or so and that’s one of the longest stretches that I’ve had in quite a while.”

(Participant 6, F)

Some of the participants not only saw stability in their health while on the modulator but noticed an improvement in their health and their lung function (FEV_1_).

“Um, although I got to say, like, physically, um… So Orkambi^®^ was great. It kept my symptoms pretty well stable. Uh, but I didn’t feel, like, a drastically noticeable difference. But when I started the Symdeko^®^, I really noticed a difference. Like my energy level increased, my pulmonary function increased, um, to the highest it’s been in five years.”

(Participant 5, M)

Participant 4 recently had an exacerbation and was hoping to stabilize his health by remaining on tezacaftor/ivacaftor.

“Um, but anyways yeah it is usually pretty typical, uh, for me because my lung function has kind of been like going up and down the past few years. But it’s kind of just been, you know, dropping down a little bit more lately. I mean the numbers have kind of just been lower recently, um, than they have in the past. So, um, I’m hoping that, you know, between the Symdeko^®^ and, you know, some other medicines that I can get things kind of like straightened out and maybe get my lung function boosted back up here.”

(Participant 4, M)

One of the participants used the imagery of the modulator being something keeping her from falling into poorer health, despite having health difficulties separate from her lung function.

“It’s a lot better. It’s a lot better like I said it’s kind of like I see it as a cushion. I feel like its support that’s keeping me… keeping me from falling. Um, and I can… I do… my lungs feel so much better on it and everything. And that definitely gives me hope. Um, so I know that as long as my lung functions are steady, um, I feel okay and I feel, you know, pretty confident. It’s just, you know, all the other phantom symptoms that we need to figure out and that’s kind of what’s dragging me down. But yeah, it’s… lung function obviously is very… very important to a lot of us with CF. You know, where we stand and everything. And I’m just very thankful that, you know, with everything that I’m battling my lung functions are at least stable. So that’s good.”

(Participant 7, F)

### Theme 2. Identity

The theme “identity” was ascertained as participants expressed that they no longer saw their diagnosis of CF as their defining feature. Participants were asked about various aspects of their lives including career, relationships, family planning, and psychological functioning. It became clear that some participants had spent their lives viewing their diagnosis of CF as their sole identifying feature and that these modulators had, in some cases, challenged those views. One of the participants said that she was able to start a career since beginning the CFTR-modulator.

“Well, I mean just, like, alone, like, um… like, the first couple of years into Orkambi^®^ I could tell a difference. But, like, I wasn’t healthy in other areas to be able to, like, hold down a job. And now I can hold down… I mean it’s a part time job, it’s not a lot of work, but I can hold it down. And I enjoy going. So I think… without Orkambi^®^ I don’t think I would be where I am right now.”

(Participant 8, F)

One participant was feeling well enough to work a full-time job since he began taking tezacaftor/ivacaftor. He expressed that his choice to disclose his diagnosis of CF to his employer was not driven by the need to ask for personal time off due to frequent hospitalizations.

“I started a new job and I don’t have a fear to tell my boss that I have cystic fibrosis anymore, right? Like I don’t have to be like: “hey I have cystic fibrosis and I might be out in the hospital and stuff like that for a while”. Because I haven’t been in the hospital in a year, right? And I’m feeling better now than I was a year ago when I came out of the hospital. So, you know, I just don’t… and I’m not saying that I would never be in the hospital again, you know, if something happens, I’ll be there. But, you know, it’s not a concern of mine. It’s just… it’s not something I feel like I need to say: “hey” …. I have told my employer I have CF but it’s not like… it’s not my identity, right? It’s not my identity anymore.”

(Participant 5, M)

Participants also talked about how CF was not the only thing they felt they had to focus on anymore.

“I think what caused a lot of stress for us was me going to the hospital. The anxiety off what’s going to happen there and how is that going to you know… how are these things going to change the outcome, you know? So, uh, I think more than anything what really calmed my anxiety was taking this job. Um, the majority of the anxiety in our relationship. And the Symdeko^®^ has been a really big part of that because now I don’t have to worry about my health and I don’t have to worry about where the money’s coming from every month, you know? So, yeah, I’m in a good space right now. A really good space.”

(Participant 5, M)

When asked what she was looking forward to about her future, one participant expressed that she hoped to live independently rather than live with a caretaker due to her diagnosis of CF.

“Number one is definitely being able to support myself. Um, I’ve always wanted to support myself and by no fault of her own my always mom used to say: ‘you’ll never be able to live by yourself’. And so of course because somebody said ‘no’ I have to be like: ‘yes I can’. So like, with this better health I am just really looking forward to being able to do that and show not just, you know, show them but I’m also, you know, I want to be independent and that I’m healthy enough to be that. And that’s what I am really hoping for. Just being able to be healthy and being able to do what I want when I need to and stuff like that.”

(Participant 1, F)

For one participant, it was difficult to not have her life defined by the life expectancy that accompanies her diagnosis of CF even while on the CFTR-modulator.

“Oh, uh, I feel happy but sad, if that makes sense? Like, um, my birthday was Monday, so I turned thirty-two and that’s good because like… like CF isn’t really much of a childhood disease anymore. Because people are living longer but like, um… obviously as you get older… like any normal person as you get older you deteriorate. But, like, there’s statistics, you know? And, like, I’m not saying, like: ‘oh I live by that statistic’, but, like, it’s hard having a number people saying: ‘well, this is the average age’. Like, I guess a normal person doesn’t have that, you know? Like, oh, well, you know: ‘average age for you to live is blah blah blah’, you know? And so, like, as I get older and closer to that number I get kind of… I do worry… it’s scary sometimes. But I mean you have to just keep on living and have hope.”

(Participant 8, F)

### Theme 3. Potentiality

The theme of “potentiality” was identified as participants expressed the desire to achieve specific goals that they did not have prior to beginning their CFTR-modulator. Participants described the potential to accomplish specific goals that they had set for themselves since beginning the CFTR-modulator. For example, one participant expressed that she now sees a potential to have a career since being on tezacaftor/ivacaftor due to less frequent hospitalizations.

“Yeah I actually have a lot more hope just to have a career at all. Because I know a lot of places they don’t want to let, have this like empty gap in employment from an employee, you know? Like: ‘oh hey by the way I have to go into the hospital for a month, hope you don’t replace me’.”

(Participant 1, F)

Even though one participant was still wary about carrying her own child, she felt that being on tezacaftor/ivacaftor would allow her to have more time with future children, leading to the possibility for her to pursue a biological pregnancy.

“I am still apprehensive about pregnancy and childbirth and all that because of the current lung function that I have. However, I almost feel like I could handle, like, one. That I could take care of and raise one of my own. Like, to where…this might sound weird or whatever, but like I said previously I didn’t want to, like, die on anybody. But now that I’m older and I have an idea about life itself… I feel like where my health is and where my mind is…and because of the hope that Symdeko^®^ and these new developing drugs have… I could raise a child to at least, you know, young teenage years and they would get enough of me to be able to handle everything that comes with life, you know what I mean?”

(Participant 1, F)

One participant expressed a goal to use his life to focus on loving his partner and raising a family, rather than focusing on maintaining his health.

“I’m looking forward to living a long life. And raising a beautiful family. And being in a committed and just a really… being with the woman that I love, right? More than anything. And I look forward to… I just look forward to what’s next. You know, life is hard, right? And you have to choose what you’re going to suffer for and I’ve already suffered for CF, you know? And I just think that this next part and my next I don’t know thirty, forty years… its’ going to be good, you know?”

(Participant 5, M)

### Theme 4. Hope

It became apparent throughout the interviews that participants were very hopeful about all aspects of their life that were addressed in the interview process. This theme of “hope” differs from the previous theme of potentiality. Potentiality refers to a participant seeing the potential of achieving a specific life goal, whereas the theme of hope refers to an overall sense of optimism since beginning the modulator. When asked how they feel when they think about the future, many participants expressed hopes around the topic of drug development and research.

“I was reading, um, catching up on CF news and Vertex expanded their division that came out with Orkambi^®^, and Kalydeco^®^, and Symdeko^®^. They doubled the size or whatever. Um, and I heard really good things about the next, um, what’s it called… the next, um, round of drugs that will be out within in a year or two…Yeah the triple, yeah. So that’s, I mean, that’s, you know, the tip of the iceberg. So increasingly hopeful for once.”

(Participant 3, M)

“Um, I think um I think I’m really looking forward to the next drug that they have with the… um…the triple. I forget what they actually named it but it’s a triple drug combination. That’s going to be a lot like Symdeko^®^ and Orkambi^®^ and those drugs, but I think that the triple is really going to be a lot more beneficial and we might see more with positive results as far as life expectancy and exacerbations go.”

(Participant 2, M)

One participant expressed that she had been positive about her future before the CFTR-modulator, but her positivity about CF research had been enhanced since she began tezacaftor/ivacaftor.

“Um, you know, I feel like I’ve always had a very positive attitude and felt that way. Um, but it is encouraging as I’ve gotten older, you know, to see the progress with the drugs and being on this one and being so stable for so long. Um, you know, I had kind of expected that, you know, that as I would get older that my health would decline. And it has a little bit but, um, I think starting the Symdeko^®^ and seeing how well it’s working and, you know, knowing that there are other drugs similar that are, you know… or even better that are being studied right now… um, just really kind of enhances that positive attitude.”

(Participant 6, F)

One participant expressed that he did not think that he would die due to his diagnosis of CF because of the advances in research.

“Um, and having… in regards to CF like, um, I don’t really think that CF is going to be the thing that kills me. I don’t think that anymore. I used to think that. But I just don’t think that anymore. Like, I just don’t think that’s my way out, you know?”

(Participant 5, M)

When asked why he believed that was the case, he continued:

“I think it’s because I’ve seen a lot of medical advances and I’ve seen the way that things are going with medicine. And we are really close to cracking the code of CF and, you know, out of all the thousands of years that people have been born with cystic fibrosis and that genetic defect… I happen to be born in a time where medical technology is at the point where it can figure out a way to fix mutations, right? Um, or get to the root cause of cystic fibrosis and that’s pretty amazing. You know, like, that is that is a blessing… that’s not… if I had been born any other time I’d be dead by now, you know? But I happen to be born now. And when I grew up… actually I grew up on the bridge, right? So when I first started they started to figure out and mapping, you know, DNA, right? And from there to now we are talking about causing, uh, causing a cure for CF. Or a pill that can manage treatments enough so all you have to do is take a pill every day, right? All you have to do is do these things. Take a few pills do a couple of treatments and you’re good.”

(Participant 5, M)

One participant discussed what she had heard from other members of the CF community forums regarding positive effects of CFTR-modulators.

“Like, as to where before it was kind of like: you’re in the dark, you’re hoping but nothing’s there yet that like, really makes you feel better. Well you have the antibiotics, but… and stuff, but there was no medicine that you take daily that makes you feel better and keep your baseline and now there is. So there’s more hope for, like, for us to be as normal as we can, if that makes sense? Like hold down jobs and have full time jobs. Or have family if that’s what you want, you know?”

(Participant 8, F)

## Discussion

**Fig. 1:**
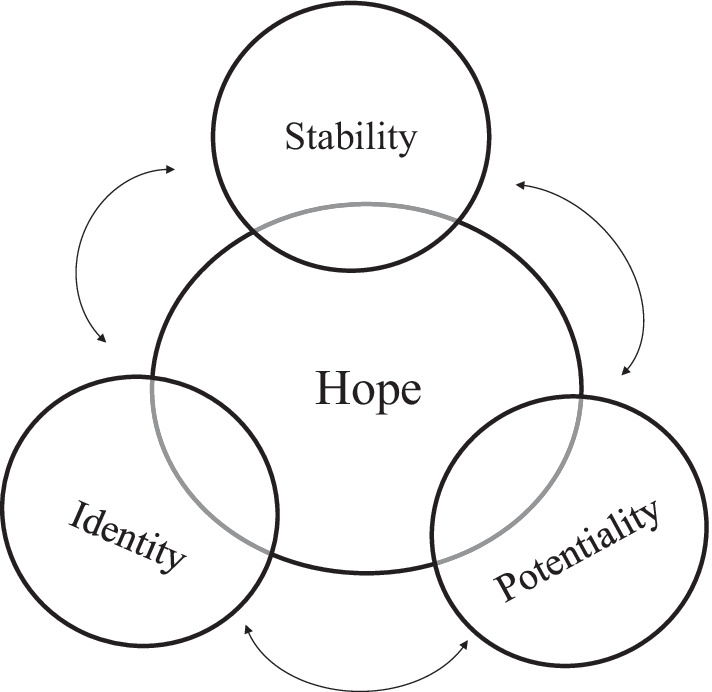
Thematic schema

The purpose of this qualitative study was to explore how the introduction of CFTR-modulators has affected individuals’ lived experiences outside of clinical health variables. Four themes emerged from the data: stability, identity, potentiality, and hope. These four themes relate to each other in an overlapping manner (Fig. 1). This thematic schema represents data provided by eight individuals in the CF community and cannot be generalized to all young adults with CF on a modulator. However, these four themes provide a springboard for future research to explore how these drugs may or may not be shifting an individual with CF’s outlook on life.

The most prominent theme throughout these interviews was the theme of “hope”, appearing at the center of the thematic schema. Participants expressed an overall feeling of hope that they mainly attributed to the CFTR-modulator that they were prescribed as well as advancing medical research. The sense of hope seen in this study ties back to the concept of psychological functioning. The three facets of psychological functioning that the semi-structured interview guide aimed to address included optimism and emotional vitality. It was clear that these participants felt optimistic and hopeful in their ability to achieve their goals since beginning the modulators. This optimism was consistent with previous literature that individuals with CF are generally hopeful and optimistic about the future [18]. The difference between the optimism found in previous literature and the optimism expressed by participants in this study is that the optimism was overwhelmingly attributed to CFTR-modulators, which were not available when the previous data were gathered. Some of the participants did bring up emotional vitality and referred to their diagnoses of depression and anxiety. An encouraging finding from these data is that some of the participants noted improvement in frequency of episodes of anxiety or depression whether it was related to financial anxiety, anxiety about life expectancy, or depression about the progressive nature of CF. For these reasons “hope” was identified as a central theme, but it also interacted with the other themes found throughout the interviews. Hope underlined how participants talked about their: careers, relationships, plans to have children, or how they viewed their outlook on life and the word “hope” was used frequently throughout the interviews.

One of the themes connected to the first theme of “hope” is the theme of “stability”. While not completely connected to the theme of “hope”, it is not entirely separate either. Some of the participants expressed feelings of stability due to the CFTR-modulator, while others expressed that these drugs gave them the hope of stability. The participants in this study defined the theme of “stability” as constant or improving health related to measurements such as: lung function (FEV_1_), current weight, frequency of pulmonary exacerbations, and number of hospital stays. These data are like the data from the CFTR-modulator phase III clinical trials showing stability in lung function and increase in weight gain. Before the introduction of CFTR-modulators, literature regarding cystic fibrosis highlighted the unpredictability of CF. Higham et al. [6] explored the hopes and fears of individuals living with CF and identified a theme of “living with unpredictable health and fear of death and dying”. Individuals in that study expressed that they viewed their disease course as unpredictable and that they expected to pass away from this disease. Similarly, a study conducted by Willis et al. [19] showed that individuals with CF expressed fear and uncertainty about the future due to the unpredictability of their health. Dissimilar from previous literature, the participants in the current study expressed control, rather than unpredictability about their future, which they credited to the CFTR-modulator. Although participants were not seeing dramatic changes in their lung function, stability at 30% or an increase to 40% in FEV_1_ was an experience these individuals had not had before taking these modulators. Although some participants did not consider themselves to be stable, they cited the CFTR-modulator as a source of hope to eventually become stable. Thus, themes of “stability” and “hope” work in tandem. Some participants felt that their stability in physical health since beginning the modulator gave them hope about their health, while others felt that the CFTR-modulators were giving them hope that their health status would eventually stabilize.

Another theme that was linked with the first theme of “hope” was the theme of “identity”. When participants were discussing their lived experiences, it became clear that cystic fibrosis played a part in how they saw themselves. It also became clear that the centrality of CF diagnosis to their sense of self or identity was shifting in some cases. It appeared that some participants were in a transition period from seeing themselves as a “patient” to seeing themselves as a “person”. Previous literature has shown that in some cases the diagnosis of CF is central to an individual’s identity. Although data on this subject have been limited in young adults with CF there are data about identity and illness centrality in adolescents with CF. A qualitative study conducted by Horky et al. [20] examined how the diagnosis of CF influenced an individual’s identity and self-image. Twenty-four male and female adolescents with CF were given video cameras and were asked to show their lives outside of CF and the hospital. Using grounded theory, the authors identified four themes in the data regarding illness centrality: CF is central, CF is compartmentalized, CF is integrated into self-image, and CF is denied. This study found that younger and healthier participants were less likely to make CF central to their identity while older and sicker individuals were more likely to make CF more central in their day-to-day lives. This seems to be consistent with the findings from this current study. “Participant 5” was able to see himself as a husband and an employee rather than solely as an individual with CF. “Participant 1” felt that she was having less frequent depressive episodes about feeling “behind” her peers in her life trajectory. Just as stability overlapped with the central theme of “hope” so does the theme of “identity”. Not all participants spoke about seeing a shift in their identity or a change in how central their illness was to their sense of self. However, some of the participants expressed hope that someday CF would not be the only thing that defines them. Rather than CF being central to their identity, these participants expressed the ability to compartmentalize being a person with CF and being a person. This demonstrates the connection between the two themes of “stability” and “identity” in this study and the fluidity between the themes found in these data.

These individuals described the potential for having the ability to attain concrete goals in the future since beginning the CFTR-modulator. These goals included but were not limited to starting a relationship or pursuing a biological family. Previous literature shows that individuals with cystic fibrosis generally have a positive outlook on their future [6, 21]. The results of this study differ from the previous literature because these eight participants were describing their future, comprised not of hopeful ideations, but of concrete goals within reach. “Participant 1” planned to have a career now that she was feeling stable on the CFTR-modulator. “Participant 8” felt she would live longer and saw herself moving forward with carrying a child now that she felt her life expectancy had been lengthened due to lumacaftor/ivacaftor. “Participant 3” felt that he would live longer being on the tezacaftor/ivacaftor and felt more comfortable pursuing a romantic relationship. Although some of the individuals interviewed did not express concrete goals related to their career, relationship, or family, they did express that they were hopeful to construct these types of goals in the future. “Participant 4” hoped that one day he would be able to be healthy enough to have a fruitful professional life by remaining on the tezacaftor/ivacaftor. This points to how the theme of “potentiality” overlaps with the central theme of “hope”. Although some participants did not express concrete goals that they wanted to achieve, they articulated hope that the CFTR-modulator would keep them healthy enough to eventually develop goals for their life.

The data from the eight participants that were interviewed in this study showed four themes related to the lived experiences of individuals with CF on CFTR-modulators. Throughout the interviews and the data analysis it was clear that individuals in this study did not experience each theme separately. Rather there was an overarching theme of “hope” at the center of the participants’ lived experience that connected all the other themes. The model that is represented in Fig. 1 shows a fluid picture of how these eight individuals described their outlook on life since beginning their CFTR-modulator. Not one of the eight participants was confined to a particular theme depicted in this model. Participants were fluid in their views, moving along the lines that connect all the themes. Some participants remained hopeful about the possibility that their health could become stable due to the modulator. These participants expressed hope that one day CF may no longer be their identifying feature if they stayed on their modulator. Some participants expressed hope to shift their focus from staying healthy to setting more aspirational life goals if they gained health from their modulator. Other participants felt that their health was stable, and their identity was no longer confined to their diagnosis of CF now that they were prescribed modulators. Different participants viewed each theme differently depending on how much hope they perceived these drugs brought to different aspects of their lives. “Participant 5” was one participant who touched on all four themes in the thematic schema and is an example of the fluidity between the themes. “Participant 5” felt that these modulators were keeping his lung function stable. From that stability he set goals for his career, felt there was less anxiety in his marriage, and set a goal to be a father. That hope and potentiality flowed into the theme of “identity”. He expressed that he never thought he would be a father, have private insurance, or hold down a job with his diagnosis of CF. This participant expressed that the hope he felt from being on tezacaftor/ivacaftor was changing how he viewed himself. He also expressed his hopes for the future of CF research and that these drugs were a first step toward a possible cure. This is an example of how each theme was touched on separately but is interconnected with an overarching theme of “hope” primarily being attributed to the modulators.

## Study limitations and strengths

This study has many limitations. The largest limitation of this study is that it was completed before the approval of elexacaftor/tezacaftor/ivacaftor. This CFTR-modulator is approved for people age six years and older with at least one F508del mutation or at least one other mutation in *CFTR* that is responsive to the medication. This triple combination therapy has proved to improve multiple health outcomes including lung function and sweat chloride concentration compared with tezacaftor/ivacaftor [22]. The data gathered in this current study are pertinent to understanding how CFTR-modulators influence psychological and social functioning of young adults with CF. However, it will be necessary to explore the lived experiences of individuals prescribed elexacaftor/tezacaftor/ivacaftor to understand how individuals with CF are adjusting in this new area of CFTR-modulators. Another limitation of this study is that the small sample size of eight individuals did not allow us to reach data saturation. The sample size was limited due to the inclusion criteria of being on a modulator for at least six months as well as the age range of 18–34 years old. A similar study needs to be conducted in a larger population to draw conclusions on if modulators affect psychosocial variables for individuals with CF. This study also only captured an instant or “snapshot” in the journey that these individuals have been on since beginning the CFTR-modulator. Perceptions of an individual’s outlook on life may not be consistent and may change after interviews were conducted. On the other hand, a strength of this study was that individuals currently experiencing an exacerbation were not excluded. This allowed the data to be more varied and not just the results of mostly healthy individuals with cystic fibrosis. Another strength was including a separate researcher to review the codebook and examine the consistency of how codes were assigned to one transcript with the primary coder.

## Practice implications

There is limited literature about the lived experiences of adults with CF since this disease was primarily considered a pediatric disease until recently. In the era of CFTR-modulators there is even less literature about the lived experiences of young adults with CF. This study’s data provides evidence that these targeted treatments may have an influence on psychological and emotional health as well as social functioning and personal goal fulfillment for individuals with CF. Thus, these preliminary data may have implications for CF care teams. The 2018 European Cystic Fibrosis Society best practice guidelines includes a section on “psychosocial support” for individuals with CF that recommends that psychosocial issues in adulthood should be addressed by the CF care team. Literature has shown that individuals with CF may not openly discuss things like employment or family planning with their CF care team and it is important that members of the team should elicit these conversations. CF care teams are encouraged to promote positive coping strategies and enlist professional psychological services where appropriate since early intervention of psychosocial issues is important [23]. These data have implications for how CF team members may approach these conversations with their patients with the new element of CFTR-modulators. Care teams who counsel adults with CF should check in with their patients and discuss if these modulators have had an influence on psychosocial areas of their lives and if so, how they are adjusting to these changes. At that point the CF care team may provide resources such as: referrals to high-risk obstetricians for women with CF deciding that they may want to pursue a pregnancy, social workers to discuss insurance changes if the individual has changed jobs, or referral to mental health professionals for individuals who feel overwhelmed by any changes in their lives brought about by the modulators.

## Research recommendations

Research focusing on the psychosocial areas of an individual’s life after beginning targeted therapies is lacking in the literature. These preliminary data show that therapies that target underlying causes of genetic disease may influence the outlook of the individuals diagnosed with these diseases. Such research may also be appropriate for other orphan diseases, which are moving in the direction of more targeted therapies such as nusinersen, which was approved for Spinal Muscular Atrophy. This qualitative study only includes the views of a few adults in the CF community. Therefore, these results cannot be generalized to all individuals with CF taking a CFTR-modulator. A study about these targeted therapies and their potential to influence psychosocial areas of an individual with CF’s life in a larger population would provide more insight into the experiences of the CF community. More current studies would also include interviews from individuals taking elexacaftor/tezacaftor/ivacaftor, which has also been shown to improve lung function and weight gain. Future studies may also focus on additional psychosocial themes apart from the ones explored in this study considering some modulators have been approved for individuals as young as six months of age. A pediatric population may generate different themes from the adult population included in this study.

## Data Availability

The datasets during and/or analyzed during the current study available from the corresponding author on reasonable request.

## Appendix 1: Interview guide

This interview is being conducted to explore your experiences of (name of CFTR-modulator). Specifically, we will be discussing your career, relationships, family planning, and psychological functioning. Your participation in this study is completely voluntary and if you feel uncomfortable answering any question, please let me know. You may choose to end this interview at any time. You will receive a $10 amazon gift card for taking the time to talk to me today.

- This interview will be recorded, is this okay?
- Would you like to continue with the interview?

*Screening questions*

- What CFTR-modulator are you currently taking?
- How long have you been taking (CFTR-modulator)?

*Demographic question*

- How old are you?

*Health*

- How is your health currently?
- How was your health before you started (CFTR-modulator)? Have there been any significant changes to your health since starting (CFTR-modulator)?

I’m going to move on to how this drug has or hasn’t impacted other areas of your life, starting with career…

## Career

1. How has your diagnosis of cystic fibrosis influenced your career path, if at all?
  - Probes: Has your health prevented you from working as many hours as you would like?
  - Probes: Have absences from work due to illness ever caused a significant disruption in your life?
2. Since starting (name of the CFTR-modulator), how has your career life changed?
  - Probes: Are you working more or less hours since starting this therapy?
  - Probes: Have you have been absent from work less or more since starting this therapy?
3. Has your satisfaction with your career changed since beginning (name of the CFTR-modulator)?
  - Probes: Have you taken on more responsibilities in your career after starting the modulators? Are you taking on additional duties, are you contributing more in meetings, are you seeking promotions?
    i. Yes: details
    ii. No: details
  - Probes: How are you feeling about your career since starting (CFTR-modulator)?

*Relationships*

1. Are you in a long-term relationship?
  - Yes: Are you married?
    i. How has having CF affected your marriage?
    ii. How has (CFTR-modulator) affected your marriage, if at all?
  - Yes: How long have you been with your partner?
    i. How has having CF affected your long-term relationship, so a relationship that has lasted a year or more?
    ii. How has (CFTR-modulator) affected your relationship, if at all?
  - No: How has having CF affected your choices about long-term relationships so a relationship that has lasted a year or more?
  - No: Has (name of CFTR-modulator) changed how you feel about long-term relationships? Do you want to get married? Do you actively want to pursue a relationship?
2. Have you noticed any changes in your social life since you began taking (CFTR-modulator)? Have you been able to keep plans with friends? Are you doing many activities outside of your home?

*Family Planning*

1. Do you have children?
  - Woman Yes: Question 2
  - Man Yes: Question 3
  - No: Question 4
2. Tell me about your CF care during your pregnancy.
3. Tell me about how you and your partner conceived.
  - Did you undergo IVF?
  - Did you adopt?
4. What are your attitudes toward starting a family?
  - Planning a family: Question 5
  - Not planning a family: has your diagnosis of CF influenced your decision not to start a family?
5. How has (CFTR-modulator) affected your attitude toward [starting a family/having more children], if at all?
  - With that in mind, tell me about any fears you have about family planning.
6. Women: What kinds of concerns do you have about your health and a pregnancy?
  - How has your attitude about this changed since you began (CFTR-modulator)?

We are moving on to the last section of the interview today…

*Psychological Functioning*

1. Have you struggled with feelings of anxiety or depression?
  - Yes: How has your life been impacted by these feelings, if at all?
    i. How have your symptoms of anxiety and/or depression been since you began (CFTR-modulator)?
2. How do you feel when you think about the future?
  - What are you looking forward to?
  - What fears do you have about the future?
  - Has your view of the future changed at all since you started (CFTR-modulator)?

- Is there anything that I didn’t ask you about?
- Is there anything you want to ask me?

Thank you for your time today.

## Abbreviations

CF: Cystic fibrosis
FEV_1_: Lung function
